# Evaluation of the Effects of Atorvastatin on the Treatment of Secondary Pulmonary Hypertension due to Chronic Obstructive Pulmonary Diseases: A Randomized Controlled Trial

**DOI:** 10.5812/ircmj.8267

**Published:** 2013-08-05

**Authors:** Seyed Ali Javad Moosavi, Hanieh Raji, Masoomeh Faghankhani, Rostam Yazdani, Mansour Esmaeili

**Affiliations:** 1 Tehran University of Medical Sciences, Tehran, IR Iran; 2 Jundishapur University of Medical Sciences, Ahvaz, IR Iran; 3 Kerman University of Medical Sciences, Kerman, IR Iran; 4 Department of Cardiology, Iranshar Hospital, Tehran, IR Iran

**Keywords:** Hypertension, Pulmonary, Chronic Obstructive Pulmonary Diseases (COPD), Statin

## Abstract

**Background:**

Since pulmonary hypertension (PH) in patients with chronic obstructive pulmonary diseases (COPD) causes poor prognosis and inflammatory process involved in PH, it is supposed that Statins with anti-inflammatory effects might be useful in the treatment of PH.

**Objectives:**

The aim of this study was to evaluate the influence of Atorvastatin on the treatment of pulmonary hypertension in patients with COPD.

**Patients and Methods:**

A registered (IRCT201108257411N1), triple-blind, randomized controlled trial was performed in Rasoule Akram hospital, Tehran, from 2009 to 2011. Forty five patients with secondary pulmonary hypertension due to COPD were recruited and randomized to two groups receiving either Atorvastatin 40 mg/d or placebo in addition to their current treatment for 6 months. The outcomes including systolic pulmonary arterial hypertension (SPAH), cardiac output (CO), right ventricular size (RVS), CRP, 6 min walk distance test (6MWD), and spirometry parameters were measured after 6 months.

**Results:**

Baseline characteristics were similar in both groups. After 6 months, pulmonary hypertension changed from 48.5 ± 6.9 to 42.9 ± 9.3 mmHg for Atorvastatin users and from 49.7 ± 11.4 to 48.2 ± 14.6 mmHg for Placebo users (P = 0.19, CI - 13.57 - 2.89), 6MWD after 6 months was 339 ± 155 meters in case group versus 340 ± 106 meters in control group (P = 0.98, CI - 92.58 - 91.15). There were no significant changes in other outcomes including CRP, RVS, CO and spirometry parameters.

**Conclusions:**

Although we found a trend towards decreasing SPAH and improving 6MWD, no statistically significant shift were detected in our outcomes due to inadequate sample size.

## 1. Background

COPD is a systemic inflammatory disease, not limited to the pulmonary system (1). The effect of cigarette smoking on the lungs has been shown to create an inflammatory process that is characterized by the recruitment and activation of inflammatory cells such as neutrophils, eosinophils, lymphocytes, and alveolar macrophages. In addition, higher baseline levels of several circulating systemic inflammatory markers, including C-reactive protein (CRP), fibrinogen, tumor necrosis factor, leukocytes, interleukin 8, and interleukin 6 in patients with COPD have been documented. Furthermore, the acute pulmonary and systemic inflammatory response induced by smoking can probably progress to a chronic persistent inflammatory process, even after the cessation of smoking (1, 2).

Pulmonary hypertension (PH) is one of the most important functional derangements in COPD and leads poor prognosis. The mechanisms leading to pulmonary hypertension in COPD have been ascribed to chronic hypoxia and loss of vascular bed from emphysematous destruction (3). In this regard, recently several studies have suggested that direct effect of smoking on the pulmonary vessels causes an increase in the level of vasoconstrictive and vasoproliferative mediators such as endothelin-1 and vascular endothelial growth factor (VEGF) in the small inter pulmonary artery. Moreover, deficiency in the production of nitric oxide (NO), created by endothelial dysfunction due to cigarette smoking, leads to deregulation of vasoconstriction, vasodilatation, and cell proliferation (4, 5). Another etiology of PH, involving arterial smooth muscles, is remodeling leading to arterial obstruction. 5-HT is considered as the molecular mediator contributing to the pathogenesis of PAH by stimulating the smooth muscle remodeling process (6).

In addition of lowering cholesterol levels and improving peripheral vascular perfusion, statins have a multiple biological effects including alteration of vascular wall structure; modulation of endothelial function (4, 5, 7) down regulation of inflammation and attenuation of oxidative stress (4, 8, 9) inhibiting muscle proliferation and causing smooth muscle cell apoptosis through inhibition of ras and rho GTPase activities, which are important for cell proliferation (10, 11); and reducing endothelin-1 mRNA expression resulting endothelin protein levels, a process that should reverse the vasoconstrictive effects of endothelin. Furthermore, statins directly up-regulate and increase the half-life of endothelial nitric oxide synthase (eNOS) mRNA and increase the ability of eNOS to generate nitric oxide, a potent vasodilator (7, 12) . Atorvastatin dose dependently inhibit 5-HT-induced pulmonary artery smooth muscle mitogenesis and migration too (13).

## 2. Objectives

Since the majority of evidence endorsing potential therapeutic benefit of statin drug class comes from a series of animal studies and there are a few data from studies in patients especially in the area of PH due to COPD, the necessity of studying such gray topic in a well-designed RCT on human seems so profitable in treating COPD patients. In our literature review, we found just one randomized clinical trial about the effect of statins on PH due to COPD (14). Therefore, we decided to perform a randomized controlled trial to investigate the effect of Atorvastatin on reducing PH of patients with COPD.

## 3. Patients and Methods

We performed a triple-blind, parallel-group, randomized controlled trial in Rasoule-Akram hospital that was conducted from 2009 to 2011. The trial protocol was approved by the institutional ethical review board at Tehran University of Medical Sciences (TUMS). The trial was registered at Iranian Registry of Clinical Trials with registration number (IRCT201108257411N1). We initially planned to recruit 60 subjects with secondary pulmonary hypertension due to COPD over approximately two years with convenience sampling method. Due to slower anticipation than recruitment, the target sample size was decreased to 45 patients. We included patients > 18 years old with SPAH and without risk factors for adverse events from this medication. Inclusion criteria were considered as Systolic pulmonary hypertension > 40mmhg, no drug history of prostanoids, statins, endothelin antagonists and phosphodiesterase, able to doing 6-min walk distance test, obstructive pattern in PFT and functional class 2,3 (NYHA). Exclusion criteria include PAH with underlying cause other than COPD; LDL < 70 mg/dl, ALT or AST > 3x upper limit normal. All participants provided written informed consent.

The criteria for diagnosis of COPD was based on American Thoracic Society standards (15), with a FEV1 (forced expiratory volume in 1 s) < 80% of the predicted values, and a FEV1/FVC (forced vital capacity) ratio < 70%. The patients who didn’t tend to attend in this study were under current treatment. Subjects were randomly assigned in two groups. We used Random Allocation software and block randomization method to determine randomized sequence and sealed opaque envelope were used for allocation concealment. Drug and placebo were nominated as two groups A and B. The patients, research analyzer, and outcome assessors (other than the Data Coordinating person) were blind until the study was completed, but current treatment was not masked. Subjects were evaluated at baseline and 6 months later. After that, one group received drug A 20 mg twice a day within 6 months and another group received drug B the same as group A.

The primary outcome was SPAH at 6 months after medication consumption. For evaluating of SPAH we used Two-dimensional and Doppler echocardiography using standard techniques on commercially available equipment with a predefined imaging protocol. To estimate systolic pulmonary artery pressure, the maximal tricuspid regurgitated jet velocity (m/s), derived from the continuous wave signal, was added to estimated right atrial pressure (RAP) (based on inferior vena cava size and its changes with inspiration) in the modified Bernoulli equation (16). One specific board-certified cardiologist did echocardiography for all participants. Secondary outcomes included 6MWD, C-reactive protein levels, cardiac output, right ventricular size, spirometry parameters such as FEV1, FVC, FEV1/FVC, oxidized low-density lipoprotein (LDL) levels, and patients’ signs and symptoms after 6 months medication consumption. Telephone assessment was done to prevent loss to follow up and assess symptoms and to encourage drug adherence.

### 3.1. Analysis

The primary analysis proceeded according to the intention-to-treat principle. Hypothesis testing for the primary and secondary end points was conducted with 2-sided α = 0.05. Continuous variables are presented as mean ± SD or median (interquartile range) and categorical variables as n (%). Baseline characteristics of the two treatment groups were compared with independent T tests. Response to Atorvastatin on SPAH, right ventricular size, cardiac output, lung function versus placebo was assessed by analysis covariance, where the response was change from baseline and the baseline value was the covariate. For non-parametric variables, we used mann-withney test. All data were analysed using SPSS software.

## 4. Results

**Figure 1.**
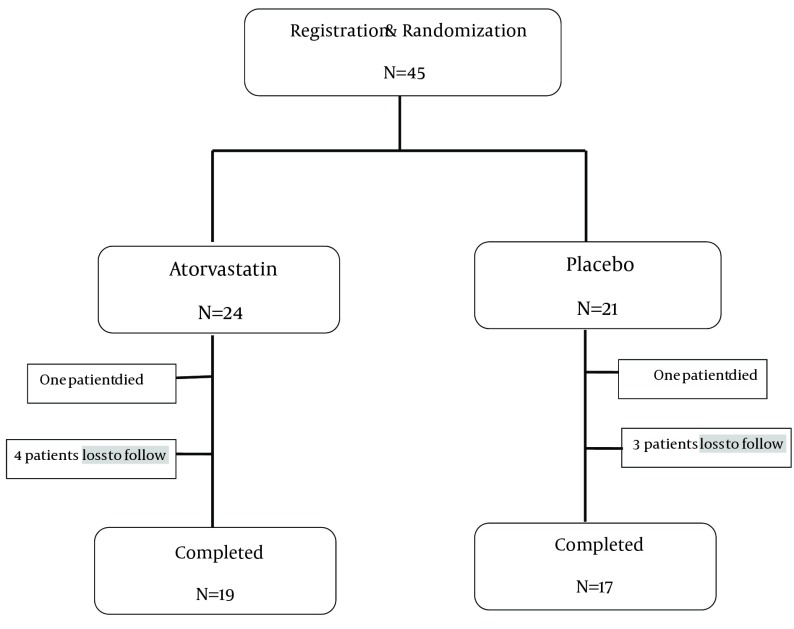
Schematic Overview of Study

Of the 45 patients who were randomized to either the Atorvastatin treatment (n = 24) or the placebo (n = 21) 36 patients completed treatment (19 in Atorvastatin group and 17 in placebo), 7 patients had incomplete data, two patients died because of deterioration of their diseases (Figure 1). Baseline demographic and clinical characteristics of patients are listed in Table 1 and Table 2. 

**Table 1.** Clinical Characteristics of the Patients at Baseline

Parameters	Atorvastatin	Placebo
**No.**	24	21
**Gender, No.**		
Male	15	13
Female	9	8
**Age, yr ± SD**	65 ± 11	68 ± 14
**Duration of underlying disease, mo**	79 ± 14	64 ± 10

**Table 2.** Clinical Outcomes After Atorvastatin Treatment or Placebo

	Baseline			6 months		
	Atorvastatin	Placebo	P Value	Atorvastatin	Placebo	P Value
**6 MWD ^a^**	238 ± 124	284 ± 100	0.17	339 ± 155	340 ± 106	0.98
**FEV1 (%)**	44 ± 25.2	43.5 ± 13.1	0.93	47.6 ± 28.4	48.6 ± 19.3	0.90
**FVC (%)**	54.7 ± 19.5	64.9 ± 18.4	0.08	62.5 ± 23.3	66.3 ± 17.9	0.58
**FEV1/VC**	57.4 ± 13.0	54.8 ± 9.9	0.46	55.9 ±15.5	57.4 ± 15.3	0.78
**Cardiac output (%)**	46.8 ± 9.7	46.6 ± 10.28	0.94	46.6 ± 5.9	46 ± 7.6	0.79
**SPAH ** ^**b**^	48.5 ± 6.9	49.7 ± 11.4	0.66	42.9 ± 9.3	48.2 ± 14.6	0.19
**RSV ** ^**c**^	2.47 ± 0.88	2.72 ± 1.25	0.46	2.40 ± 1.02	2.33 ± 0.84	0.82
**LDL (mg/dl)**	109 ± 33	104 ± 31	0.66	86 ± 21	111 ± 33	0.01

^a^ 6 Min Walk Distance (meter)

^b^ Systolic Pulmonary Arterial Hypertension (mmHg)

^c^ Right Size Ventricle (cm)

The two groups were well balanced with respect to these characteristics. Changes in clinical outcomes after Atorvastatin treatment are listed in Table 2. At 6 months, atorvastatin treatment in patients with SPAH, as compared with placebo, had no statistically significant influence on the pulmonary hypertension. A trend toward a decrease in SPAH was observed in the statin group at the end of 6 months in comparison with baseline which has shown that this decrease was statistically significant( from 48.5 ± 6.9 to 42.9 ± 9.3 mmHg, P = 0.003). The difference in 6-minute walk distance at 6 months in favor of Atorvastatin (from 238 ± 124 to 339 ± 155 meters, P = 0.003) that in comparison of placebo was not statistically significant Table 2. There was no significant difference in hepatic enzymes between the two treatment groups. No significant difference in the CRP was found between the two groups at baseline or 6 months. Atorvastatin was very well-tolerated by all patients, and none of them had any significant subjective side effects, such as abnormal levels of liver and muscle enzymes. Patient compliance with the treatment was confirmed by the significant effects on blood lipids. Baseline LDL cholesterol levels were 109 ± 33 mg/dl in the statin and 104 ± 31 mg/dl in the placebo-treated groups (P = 0.75). At 24 weeks these were 86 ± 21 and 111 ± 33 mg/dl (P = 0.01), respectively, indicating good compliance with statin treatment. 

## 5. Discussion

There is a lot of interest in the use of statins for the treatment of pulmonary arterial hypertension (PAH). Statins have recently come under evaluation for the treatment of PAH. The potential role of statins in treating COPD is controversial. The current evidence shows that statins have an effective influence on outcomes in patients with COPD. Despite these hopeful results, in all researches up to this time, there are many limitations that would be in mind. The effective dose, interval and duration of administration and effect of various statins will be needed to appear the advantage of them, in target population is unclear. Although these are new evidence but insufficient to vindicate a clinical indication for statin therapy in patients with COPD regardless of protection of them on cardiovascular system. This information support that new RCT should be designed to approved these advantages. A positive effect on outcomes, such as exacerbation, hospitalization and mortality rates in patients with COPD, potentially could have a large beneficial effect on the individual, social, and economic consequences of this disease.

To our knowledge the present study has been one of the first randomized controlled trials to evaluate the effects of statin treatment on SPAH due to COPD. In this study we found no statistically improvement in the atorvastatin group compared to the placebo group in SPAH, lung function, CRP, 6MWT in COPD patients with pulmonary hypertension. There are several limitations to this study (1) The Majority of our patients were not stable who referred to hospital with exacerbation of symptoms and they received active treatment for COPD in addition to atorvastatin or placebo which might reduce the potential to detect a beneficial effects of atorvastatin in a relatively short study period (2) This was a short-term study that was not designed to evaluate on survival advantage (3) We used a moderate dose of atorvastatin and probably in higher dose the results could be significant (4) Low sample size was one of our study limitations. However, the negative results obtained in our study do not exclude the potential benefits of statin use in COPD patients with SPAH.

The only RCT of statin use in patients with COPD and PAH was done by Lee et al. that evaluated the hypothesis whether statins improve hemodynamic in COPD patients with PH by affecting ET-1 synthesis. A total of 53 patients with stable COPD [FEV1 (forced expiratory volume in 1 s) 56% predicted] and PH (defined as a systolic PAP > 35 mmHg by echocardiography) were randomized to receive either pravastatin or placebo in a double-blind group for 6 months. The primary outcome was exercise capacity, tested using a Naughton stress test treadmill protocol, and was significantly improved in the pravastatin-treated group in comparison with placebo group. Systolic PAP decreased significantly from 47 to 40 mmHg, and the Borg dyspnoea index was also improved. The main conclusion of the authors was that “pravastatin significantly improved exercise tolerance, and decreased PH and dyspnoea during exercise, probably by inhibiting ET-1 synthesis in COPD patients with PH (14). The design of this study was similar to our study and difference in results may be due to lower sample size in our study.

Peter N. Kao evaluated the safety and efficacy of simvastatin for treatment of patients with PAH in an open-label observational study performed on sixteen patients with primary and secondary causes of PAH. Simvastatin was prescribed at 20 to 80 mg/d and continued in the absence of adverse effects. Serial measurements of 6-min walk (6MW) performance, hemodynamics, and echocardiographic estimates of right ventricular systolic pressures (RVSPs) were recorded on each patient. Improvements in 6MW performance, cardiac output, or decreases in RVSP that may be attributable to simvastatin treatment, were demonstrated (11). Martin R and his associated assessed the therapeutic value of simvastatin in patients with pulmonary arterial hypertension (PAH). Forty-two patients with PAH were randomized to receive either simvastatin (80 mg/d) or placebo in addition to current treatment for 6 months, and thereafter offered open-label simvastatin. Change in RV mass, was assessed by cardiac magnetic resonance (CMR) was primary outcome. At 6 months, RV mass and N-terminal pro–B-type natriuretic peptide (NT-proBNP) levels decreased in the stain users compared with non-statin users. There were no significant changes in other outcome measures (including 6-minute walk test, cardiac index, and circulating cytokines). From 6 to 12 months, both RV mass and NT-proBNP increased toward baseline values in 16 patients with active treatment who continued with simvastatin, however these values remained stable in 18 patients whose placebo switched to simvastatin (17).

Przemysław K and his colleagues evaluated the influence of simvastatin on selected inflammatory markers in patients with COPD. The study shows that a 3-month treatment with simvastatin does not reduce circulating inflammatory markers in the patients (18). Blamoun A.I and et al. studied in a retrospective cohort study of 185 patients with COPD exacerbation, with a 1-year follow-up. Outcomes assessed were repeat hospitalization and intubation rate for COPD exacerbations. They found the statin group had fewer episodes of exacerbation and required less intubation than the subjects not receiving statins (19). In another study by Soyseth et al. has been found that the mortality among COPD patients after discharge from hospital was lower among those who were taking a statin compared with those who did not (20). The retrospective cohort study by van Gestel et al. involved 3,371 patients with peripheral arterial disease undergoing peripheral vascular surgery. Subgroup analysis of patients with COPD showed that statin users had lower short-term mortality (30-day) and long-term mortality (10-year) than non-statin users (1, 21).

In another study, Reed et al. evaluated 112 patients with severe COPD regarding clinical characteristics, pulmonary function, cardiac catheterization findings, and medical co morbidities in a cross-sectional study. They compared these outcomes between 30% statin users and 70% non-statin users. They found that statin use was associated with a 4.2 mmHg (95% CI: 2 to 6.4, P = < 0.001) lower PAWP and a 2.6 mmHg (95% CI: 0.3 to 4.9, P = 0.03) reduction in mean PAP independent of PAWP (22). In a summary, we found statistically non-significant improvement in SPAH in statin users with no adverse effect of the drug due to the limitations of study. Considering findings of previous studies and such a positive trend in our study towards statin benefits in PAH treatment of COPD patients, Although we cannot come in conclusion that the addition of statins to the treatment of patients with PAH as an adjuvant therapy to current treatment for PAH is safe and well tolerated with definite advantages, we can suggest that more developed RCTs with adequate sample size may prove such effect of statins and open a new window for physicians towards COPD treatment and for patients towards higher life quality. In conclusion, Atorvastatin treatment in patients with PH due to COPD had no statistically significant influence on the PH measured during the observation. A trend towards a decrease in PH level was observed in the statin group, but it did not reach statistical significance. An insignificant decrease in 6MWD was observed in patients receiving Atorvastatin.
